# 不同治疗对肺癌患者血液循环游离DNA检测*EGFR*基因突变的影响及预后分析

**DOI:** 10.3779/j.issn.1009-3419.2018.05.06

**Published:** 2018-05-20

**Authors:** 飞 苏, 可 郑, 祎云 付, 倩 吴, 源 唐, 威亚 王, 莉莉 蒋

**Affiliations:** 610041 成都，四川大学华西医院病理科 Department of Pathology, West China Hospital of Sichuan University, Chengdou 610041, China

**Keywords:** 肺腺癌, 循环游离DNA, EGFR, T790M突变, 预后, Lung neoplasms, Circulating cell-free DNA, EGFR, T790M, Prognosis

## Abstract

**背景与目的:**

表皮生长因子受体（epidermal growth factor receptor, *EGFR*）基因突变与肺腺癌患者TKI靶向治疗疗效和预后密切相关，常规组织分析其突变状态有诸多局限。本研究旨在探讨非小细胞肺癌（non-small cell lung cancer, NSCLC）患者血液循环游离DNA（cell-free DNA, cfDNA）检测*EGFR*基因突变在治疗前、传统化疗以及靶向治疗后的表达差异。分析血液cfDNA是否能准确检测*EGFR*基因突变并监测耐药基因T790M的变化，以及TKI在靶向治疗患者中的预后价值。

**方法:**

应用ARMS（amplification refractory mutation system）法检测107例（50例治疗前、29例传统化疗和28例靶向治疗）肺癌患者配对血液和肿瘤组织样本的*EGFR*基因突变并比较其表达差异；计算检测的一致性、敏感性和特异性；分析血检对靶向治疗患者的预后价值。

**结果:**

血浆cfDNA检测*EGFR*总突变率在107例肺癌患者中为56%（60例），而配对肿瘤组织样本检出率为77.6%（83例）。一一配对比较发现两者总体一致率为68.2%。血检的敏感性是72.3%，特异性为100%。依据治疗状态分组后发现治疗前组患者血液和肿瘤组织样本的检测一致率最高（74%, 37/50），而靶向组一致率最低（57.1%, 16/28），提示靶向治疗改变血浆cfDNA中*EGFR*基因状态。具体分析靶向组不一致病例发现50%新检出含T790M的双突变，提示靶向治疗后耐药基因出现。生存分析证实血检含T790M双突变组的无进展生存期（progression-free survival, PFS）和总生存期（overall survival, OS）均显著低于无T790M突变组。

**结论:**

应用ARMS法检测血液循环游离DNA（circulating cell-free DNA, cfDNA）的*EGFR*基因突变是一种特异性高、敏感性好的检测方法。适用于治疗前晚期肺癌患者的*EGFR*基因突变状态检测。同时，适用于靶向治疗后监测T790M耐药突变状态及预测患者预后。

## Abstract (en)

**Background and objective:**

Epidermal growth factor receptor (*EGFR*) gene mutation is closely related to the EGFR-TKI target treatment and prognosis of lung adenocarcinoma patients. The mutation status of *EGFR* is limited by tissue detection. The purpose of this study was to investigate the difference of *EGFR* mutants in plasmacirculating cell-free DNA (cfDNA) obtained from patients with non-small cell lung cancer (NSCLC) in three groups: pre-therapy, after traditional chemotherapy and targeted therapy. The aim of this study was to analyze whether the plasma cfDNA could effectively determine the *EGFR* mutations and monitor the drug resistant gene T790M, as well as its prognostic prediction value in patients with targeted therapy.

**Methods:**

ARMS (amplification refractory mutation system)-PCR was used to detect *EGFR* mutations in 107 (50 of pre-therapy, 29 after traditional chemotherapy and 28 after targeted therapy) cases of paired plasma and tumor tissue specimens, followed by comparing their concordance. The sensitivity, specificity and the prognostic value of plasma cfDNA detection were also observed.

**Results:**

The total rate of *EGFR* mutation was 56% (60/107) in all plasma samples and 77.6% (83/107) in corresponding tumor tissues. Completely the same mutants and wild-type EGFR were found in 68.2% cases of paired specimens. The sensitivity of plasma cfDNA detection was 72.3% and the specificity was up to 100%. Patients were sub-categorized according to therapy. The results showed that the highest consistent rate of cfDNA and tumor tissues was found in the group of pre-therapy (74%, 37/50). Whereas, the lowest consistent rate was observed in the targeted therapy group (57.1%, 16/28). It indicated that the targeted treatment could change the EGFR status in plasma cfDNA. Further analyses on inconsistent cases in this group revealed that 50% of them were compound *EGFR* mutations with T790M. Thereby, it suggested that targeted therapy might induce the emergence of drug resistance gene T790M. This speculation was confirmed by survival analyses. Based on plasma cfDNA results, patients with T790M mutant had significantly worse progression-free survival (PFS) and overall survival (OS).

**Conclusion:**

For EGFR testing, ARMS-PCR on plasma cfDNA is a promising methodology with the highest specificity and effective sensitivity. It is useful for EGFR testing in patients before treatment, especially the late-stage patients. Simultaneously, plasma cfDNA could be used to monitor the drug resistant mutation, T790M status and predict prognosis after targeted therapy.

肺癌是世界范围内致死率最高的恶性肿瘤^[1]^，也是中国最常见的恶性肿瘤之一。中国每年新发肺癌病例60.95万，居恶性肿瘤首位^[2]^。其中，约80%为非小细胞肺癌（non-small cell lung cancer, NSCLC）患者。75%患者确诊时已是晚期，失去手术机会^[3]^。目前，表皮生长因子受体酪氨酸激酶抑制剂（epidermal growth factor receptor-tyrosine kinase inhibitors, EGFR-TKIs）已是携带*EGFR*基因敏感突变的NSCLC患者的一线治疗用药^[4-8]^。及时、全面了解*EGFR*突变对NSCLC患者的治疗及预后具有重要指导意义。

通过活检或术后肿瘤组织进行*EGFR*基因检测是现行金标准。但是，临床实践中存在诸多局限，如：肿瘤组织获取困难、难以重复取材、以及肿瘤异质性等。液体活检是指以非侵入性或微侵入性方法，通过对痰液、血液、尿液等体液中的循环肿瘤细胞（circulating tumor cell, CTC）、循环肿瘤DNA（cell-free DNA, ctDNA）、循环游离DNA（circulating cell-free DNA, cfDNA）和外泌体等进行检测，从而获取肿瘤组织生物学信息的技术^[9]^。与组织活检相比，液体活检具有微创、可重复、患者接受度高等特点。cfDNA是患者血浆中游离的自身DNA片段，由正常及肿瘤细胞坏死、凋亡或主动分泌进入外周血循环，携带生物学信息与原发肿瘤具有高度一致性^[10]^。当肿瘤组织难以获取时，血浆cfDNA检测是*EGFR*突变分析合适的替代选择^[11]^。对第一代EGFR-TKIs耐药的肺癌患者中，约50%出现T790M突变。这种耐药突变可以存在于原发肿瘤中，更多是出现在治疗后二次活检样本。显然，血检在随访、监测方面更具有可操作性。

本研究收集肺癌患者配对的血液及肿瘤组织样本进行*EGFR*基因检测分析，旨在探讨NSCLC患者血液循环cfDNA检测*EGFR*基因突变的敏感性和特异性，分析其临床应用以及预后指导价值，为临床常规应用提供依据。

## 1 材料与方法

### 1.1 病例资料和样本收集

收集2014年12月-2017年4月四川大学华西医院病理科107例肺腺癌患者一一配对的血液和肿瘤组织样本，所有配对的肿瘤组织样本均取自辅助治疗前。收集并分析患者临床病理特征，包括：性别、年龄、吸烟史、肿瘤类型、分期、治疗方式等资料。入组标准：①病例资料完整；②肝、肾功能及血常规无明显异常，无其他并发恶性肿瘤或器官功能障碍性疾病。③其中，血液样本包括3组：治疗前血液样本50例，取患者治疗前的肿瘤组织标本同时收集血液标本，包括活检组织[计算机断层扫描（computed tomography, CT）引导下经皮肺穿刺和纤维支气管镜活检]38例和手术切除样本12例。传统化疗后血液样本29例，收集患者化疗前的活检肿瘤组织样本和化疗五期后血液样本；靶向治疗后血液样本28例，收集患者靶向治疗前活检肿瘤组织样本；对靶向治疗的28例腺癌患者全部进行随访：随访时间：2014年12月-2017年12月；随访中位时间19.5个月，随访间隔时间3个月；随访方式：门诊随访及电话随访；随访内容包括临床症状，体格检查淋巴结情况、肿瘤标志物水平[癌胚抗原（carcino-embryonic antigen, CEA）、细胞角蛋白19片段（cytokeratin-19-fragment, CYFRA21-1）、神经元特异性烯醇化酶（neuron-specific enolase, NSE）等]、影像学检查资料等；根据随访情况，判断为病情进展时，收集患者血液样本；其靶向治疗平均时间为12个月。

### 1.2 组织样本诊断及DNA的提取

107例配对组织样本均由两位资深病理医师（蒋莉莉副主任医师，王威亚副主任医师）诊断组织学亚型，必要时行免疫组化协助确诊[广谱细胞角蛋白（pan-cytokeratin, PCK）、细胞角蛋白7（cytokeratin 7, CK7）、甲状腺转录因子（thyroid transcription factor-1, TTF-1）、天门冬氨酸蛋白酶A（noval aspartic proteinase pepsin family A, Napsin-A）、CK5/6、P63、P40、CD56、嗜铬素A（chromogranin A, CgA）、突触素（synaptophysin, Syn）和ki-67]。同时，选择肿瘤细胞占比10%以上的蜡块提取DNA。肿瘤组织DNA的提取采用商品化试剂盒（DNA FFPE Tissue Kit, QIAGEN），样本浓度和纯度使用紫外分光光度计检测，确保光密度（optical density, OD）值在1.8-2.0（A_260_/A_280_）之间。合格DNA样本于-20 ℃环境保存，检测时稀释至2 ng/μL备用。

### 1.3 血液样本的收集及DNA的提取

107例血液样本均于清晨空腹抽取静脉血10 mL置于K2E（EDTA）抗凝采血管（BD）中，并于30 min内处理：2, 000 *g*离心10 min后将上层血浆移至新离心管，再重复2, 000 *g*离心10 min提纯；获得的4 mL-5 mL上清血浆标本保存在-80 ℃低温冰箱，24 h内完成cfDNA提取（血液DNA提取分离试剂盒，艾德，中国），并于-20 ℃保存待检。

### 1.4 *EGFR*基因突变检测

成功提取DNA后在3 d内进行ARMS法（ABI 7500 real-time PCR仪，美国应用生物公司）检测*EGFR*基因突变，包括18-21号外显子共29个突变热点（ADx-ARMS，艾德，YZB/国6377-2014）。每次实验均设各突变阴性、阳性对照孔及空白对照孔，反应参数依据试剂盒说明书设定。根据说明书判定标准对PCR反应扩增曲线及CT值进行分析。

### 1.5 统计方法

数据采用IBM SPSS 19.0软件进行分析和处理，计量资料以均数±标准差（Mean±SD）表示，采用方差分析；计数资料用率（%）表示，应用*χ*^2^检验对计数资料进行分析。应用*Kaplan-Meier*曲线进行生存分析，组间曲线比较采用*Log-rank*检验，*P* < 0.05为差异有统计学意义。

## 2 结果

### 2.1 临床病理特征

本组107例肺癌患者的临床病理特征见表 1。其中男女比例为1:1.4；平均年龄58岁（29岁-81岁）；大部分患者无既往吸烟史；超过90%患者属于肺癌晚期（Ⅲ期18例，Ⅳ期80例）。组织学类型以低分化腺癌为主。

**1:** 111例肺癌患者配对血液和肿瘤组织样本的*EGFR*突变分析 Analysis of *EGFR* mutation in paired blood and tumor tissue samples from 107 patients with lung cancer [*n* (%)]

Clinical parameter	No. of sample	Blood				Tissue		
		Mutant	Wild type	*P*		Mutant	Wild type	*P*
Gender				0.095				0.172
Male	45	21 (46.7)	24 (53.3)			32 (71.1)	13 (28.9)	
Female	62	39 (62.9)	23 (37.1)			51 (82.3)	11 (17.7)	
Age (yr)				0.913				0.181
< 60	54	30 (55.6)	24 (44.4)			39 (72.2)	15 (27.8)	
≥60	53	30 (56.6)	23 (43.4)			44 (83.0)	9 (17.0)	
Smoking history				0.988				0.791
Yes	16	9 (56.3)	7 (43.7)			12 (75.0)	4 (25.0)	
No	91	51 (56.0)	40 (44.0)			71 (78.0)	20 (22.0)	
Stages								
I	2	1 (50.0)	1 (50.0)			2 (100.0)	0	
Ⅱ	7	1 (14.3)	6 (85.7)			2 (28.6)	5 (71.4)	
Ⅲ	18	4 (22.2)	14 (77.8)	0.032^a^		10 (55.6)	8 (44.4)	0.013^a^
Ⅳ	80	54 (67.5)	26 (32.5)	0.000^b^		69 (86.3)	11 (13.7)	0.003^b^
Differentiation				0.262				0.165
High	34	18 (52.9)	16 (47.1)			23 (67.6)	11 (32.4)	
Middle	19	8 (42.1)	11 (57.9)			17 (89.5)	2 (10.5)	
Low	54	34 (63.0)	20 (37.0)			43 (79.6)	11 (20.4)	
^a^Comparison of mutation rates in stage Ⅰ, Ⅱ and Ⅲ, Ⅳ; ^b^comparison of mutation rates in patients with stage Ⅲ and Ⅳ. EGFR: epidermal growth factor receptor.								

配对107例肺癌患者的血液样本包括3种情况：①未经辅助治疗（治疗前组）患者样本50例（46.7%）；②传统化学治疗后（化疗组）患者样本29例（27.1%），包括AC（培美曲塞+卡铂）化疗12例，AP（普莱乐+顺铂）化疗17例；③靶向治疗（靶向组）患者样本28例（26.2%），其中以吉非替尼治疗24例，特罗凯治疗2例，以及凯美纳、ADZ9291治疗患者各1例。

### 2.2 *EGFR*总体突变率与类型

107例配对血液和肿瘤组织样本均成功进行*EGFR*基因突变分析（ARMS法），与临床特征分析见表 1。统计分析结果显示，血液和组织*EGFR*突变均在晚期肺癌患者，尤其是Ⅳ期患者中显著增高（67.5%和87.5%，*P* < 0.001，*P*=0.002）；在女性腺癌患者中突变率较高，但差异无统计学意义。

血浆cfDNA检出*EGFR*突变60例，总体突变率为56.1%。突变类型以19号外显子缺失（19 deletion, 19Del）（27例，45%）和21号外显子突变（L858R）（22例，36.7%）为主；余11例均为双重*EGFR*突变（compound *EGFR* mutations），包括：19Del T790M双突变7例（11.7%），L858R T790M双突变2例，G719X L861Q双突变和19Del L858R双突变各1例。

配对肿瘤组织样本*EGFR*突变检出率略高于血液样本，达77.6%（83例）。突变类型包括19Del 44例（53.0%）、L858R 30例（36.1%）以及少见突变18号外显子（G719X）突变和20号外显子插入突变（20-INS）各1例。另检出双重*EGFR*突变7例，包括：19Del T790M双突变4例（4.8%），19Del L858R双突变2例和G719X、S768I各1例。

### 2.3 血液与肿瘤组织*EGFR*突变结果比较

一一配对比较血液和肿瘤组织样本的*EGFR*突变总体情况，完全一致样本共73例（68.2%），包括49例*EGFR*突变型和24例野生型。检测一致的突变类型包括19Del 27例（55.1%），L858R 21例（42.9%），G719X L861Q双突变1例。不一致病例34例（表 2），主要表现为假阴性：血液样本呈野生型而组织为*EGFR*突变型（23例，67.6%）。余11例不一致者均属于*EGFR*突变类型差异，主要集中在血液样本T790M的检出率更高（9例，81.8%）。同时，没有出现血液样本假阳性病例。因此，血液cfDNA检测*EGFR*基因突变的敏感性为72.3%，特异性为100%。血液检测*EGFR*突变型和野生型的一致率分别为81.7%、48.9%，差异有统计学意义（*P* < 0.001），提示血液检测*EGFR*突变阳性结果可能更可靠。19Del和L858R突变的一致率分别为61.4%和70%，两者差异无统计学意义（*P* > 0.05），提示血液检测两种突变类型均较可靠。

**2:** 配对血液与肿瘤组织样本*EGFR*基因检测结果不一致病例 Inconsistent results of *EGFR* mutation detection in matched blood and tumor tissue specimen

Group	Blood	Tissue	No. of sample
Pre-therapy group (*n*=13)	19Del T790M	19Del	2
	L858R T790M	L858R	1
	L858R	19Del L858R	1
	19Del L858R	L858R	1
	Wild type	20-INS	1
	Wild type	L858R	2
	Wild type	19Del	5
Targeted therapy group (*n*=12)	19Del T790M	19Del	5
	L858R T790M	L858R	1
	Wild type	19Del	1
	Wild type	L858R	3
	Wild type	19Del L858R	1
	Wild type	19Del T790M	1
Chemotherapy group (*n*=9)	Wild type	G719X	1
	Wild type	L858R	1
	Wild type	19Del	4
	Wild type	19Del T790M	3

107例血液样本依据治疗状态分为3组（表 2）。①50例治疗前患者血液样本检出*EGFR*突变31例（62%），配对肿瘤组织*EGFR*突变39例（78%）。两者*EGFR*突变率差异无统计学意义（*P*=0.081）。配对比较结果发现完全一致样本37例（74%，3组中最高）。其中19Del突变12例，L858R突变13例，G719X L861Q双突变1例和野生型11例。检测结果不一致样本13例，同样集中在假阴性病例（9例，69.2%）。有趣的是，3例患者治疗前血检即发现含T790M双突变，而组织检测阴性。②靶向组患者28例。血液检出*EGFR*突变19例（67.9%），配对组织25例（89.3%），两者差异无统计学意义（*P*=0.051）。与配对治疗前肿瘤组织样本比较，检测结果一致样本16例（57.1%，3组最低），其中19Del突变样本10例，L858R突变样本3例，野生型3例。具体分析12例不一致病例发现：假阴性和检出含T790M双突变病例各占一半（表 2）。③化疗组患者29例。血液检出*EGFR*突变10例（34.5%，3组最低），配对治疗前肿瘤组织检出*EGFR*突变19例（65.5%），差异有统计学意义（*P*=0.018）。两者检测结果一致样本20例（65.6%），包括19Del突变5例，L858R突变5例和野生型10例。重要的是9例不一致样本全部属于假阴性（表 2），提示传统化疗后血液*EGFR*基因突变检出率显著降低。

### 2.4 *EGFR*基因检测与靶向治疗预后分析

28例肺癌患者经靶向治疗后检测血液cfDNA了解*EGFR*基因状态，并全部随访；依据血检结果分为含T790M双突变组（6例）和无T790M突变组（13例）：含T790M双突变组患者平均生存期17.5个月，无T790M突变组平均生存期29.6个月，两者差异具有统计学意义（*P* < 0.05）。生存分析如图 1所示，无T790M突变组的无进展生存期（progression-free survival, PFS）和总生存期（overall survival, OS）均比含T790M双突变组患者显著延长。进一步分层分析发现，无论男性还是女性，无论 < 60岁组还是≥60岁组，含T790M双突变组预后均较差（*P*=0.005）。

**1:**
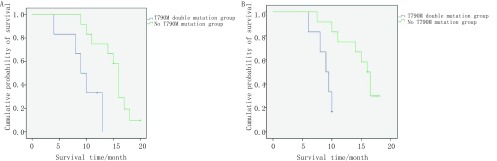
T790M双突变组和无T790M突变组患者预后分析。A：T790M双突变组和无T790M突变组PFS比较（*P*=0.006）；B：T790M双突变组和无T790M突变组OS比较（*P*=0.003）。 Prognostic analysis of patients with T790M double mutation and no T790M mutation. A: Comparison of progression-free survival (PFS) time between T790M double mutation group and T790M free mutation group survival (*P*=0.006); B: Comparison of overall survival (OS) time between T790M double mutation group and no T790M mutation group (*P*=0.003).

进一步细分血检结果为19Del突变组、L858R突变组和含T790M双突变组进行预后分析，发现19Del突变组患者平均生存期为33.2个月，L858R突变组平均生存时间19个月，含T790M双突变患者平均生存时间为17.5个月。三者的PFS、OS差异均具有统计学意义（图 2）。但两两对比分析发现，19Del突变患者较L858R突变患者和含T790M双突变患者靶向治疗后PFS和OS明显延长，而L858R突变与含T790M双突变患者预后差异无统计学意义。

**2:**
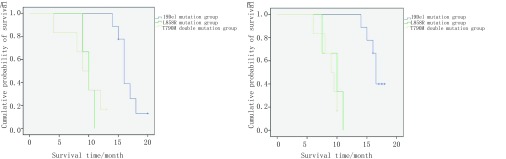
19Del突变组、L858R突变组和T790M双突变组患者预后分析。A：19Del突变组、L858R突变组和T790M双突变组PFS比较（*P*=0.001）；B：19Del突变组、L858R突变组和含T790M双突变组OS比较（*P* < 0.001）。 Prognostic analysis of 19Del mutation group, L858R mutation group and T790M double mutation group. A: Comparison of PFS between the 19Del mutation group and the T790M double mutation group (*P*=0.001); B: Comparison of OS between the 19Del mutation group and the T790M double mutation group (*P* < 0.001).

## 3 讨论

分子靶向治疗已成为肺癌患者常规治疗的重要部分。临床病理多采用侵入性方法，如活检或手术获取的组织进行*EGFR*基因突变检测^[12]^。其优点毋庸置疑，但缺点同样不可忽视。一方面，这些侵入性方法都难以重复取材并伴有一定风险^[13]^，并发症可达17%^[14]^。另一方面，大部分NSCLC患者确诊时已是晚期，近1/3患者无法获得适用于EGFR检测的组织样本^[15]^。此时，血浆cfDNA是肺癌患者*EGFR*突变检测的一种有效的替代选择。近年来，关于这方面的研究呈井喷式发展。本研究对一组配对血液和肿瘤组织样本的*EGFR*基因进行检测，比其表达差异，分析血液cfDNA是否能准确检测*EGFR*基因突变并监测耐药基因T790M的变化，指导患者个体化精准治疗。

本研究发现应用ARMS-PCR法检测血液cfDNA的*EGFR*突变总体一致率为68.2%，方法敏感性为72.3%，特异性为100%，比以往的研究^[16]^更佳。更有意义的是，我们在治疗前组获得血检和组织检测最高一致率（74%），最低一致率出现在靶向组（57.1%）。同时，化疗组中发现血液*EGFR*突变检出率明显低于组织（*P*=0.018），这与以往报道化疗后*EGFR*突变率降低^[17]^相符。这些数据均提示术后辅助治疗（传统化疗和靶向治疗）可以影响血浆cfDNA构成，为了解残余肿瘤状态提供依据。

进一步分析血检和组织检测不一致病例发现：①治疗前组主要表现为假阴性（57.1%, 8/14），提示血检作为无法获得原发肿瘤组织的替代选择时应谨慎解释阴性结果。②靶向组不一致病例一半为假阴性，一半为含T790M双突变（表 2）。同时，生存分析发现含T790M双突变组无论在PFS还是OS均差于无突变组（图 1）。因此，血液cfDNA检测*EGFR*突变能更好地反映治疗现状并提示预后。另一方面，Camidge等^[18]^研究发现接近一半的患者在接受连续的EGFR-TKI治疗后出现继发性T790M突变。针对耐药突变T790M的第三代EGFR-TKI已进入视野，及时检测以及监测用药后T790M状态成为临床关注新热点^[19, 20]^。本研究同样提示血液cfDNA监测T790M突变也可作为选择靶向药物的指针。③化疗组不一致病例全部属于血检野生型而治疗前原发肿瘤组织为*EGFR*突变型，提示血检应用于传统化疗后的随访监测可能作用局限。

治疗前组有3例血液含T790M双突变而配对肿瘤组织中阴性的病例以及1个血检双突变（19Del、L858R）而组织单突变（L858R）病例。我们分析出现这种情况可能的原因包括：组织活检取材过程中存在选择性偏差，因此局部肿瘤组织与外周血所携带生物学信息不一致^[21]^；肿瘤组织的异质性也可能导致与血浆游离DNA信息差异；呈T790M突变的肿瘤细胞亚群均有更高增值指数，易于侵袭性生长或坏死并释放DNA入血。因此，本研究提示血浆cfDNA比活检小组织更能全面反映肿瘤驱动基因状态，均化组织异质性造成的检测偏差，为治疗提供更加全面的信息。

分析不同突变类型经靶向治疗后的无进展生存期和总生存期，并对年龄、性别等方面进行分层对比。结果显示无T790M突变患者生存期均较突变患者长（PFS及OS），并且不受性别和年龄影响。经靶向治疗的不同*EGFR*突变类型患者中，19Del突变预后最佳，较L858R和含T790M双突变患者PFS和OS明显延长；而L858R突变型较T790M双突变型预后稍好，但差异无统计学意义。与本研究一致，有文献^[22-25]^报道19Del突变患者靶向治疗生存期较L858R突变患者更长。

综上所述，应用ARMS法检测血浆cfDNA的*EGFR*基因突变是一种特异性高、敏感性好的检测方法，适用于晚期治疗前肺癌患者的靶向检测，是无法获得组织样本时合适的替代物。其优势在于能全面反映肿瘤*EGFR*基因状态，减少组织异质性造成的检测偏倚。同时，适用于应用靶向治疗后：①监测T790M耐药突变状态，实时反映治疗效果，指导用药；②具有预后价值等。其局限性在于：①需谨慎解释阴性结果；②传统化疗后可能出现假阴性。全面了解这一检测方法的优缺点，利于临床选择开展相应工作及正确解读结果。
